# Measuring the Effectiveness of Conservation: A Novel Framework to Quantify the Benefits of Sage-Grouse Conservation Policy and Easements in Wyoming

**DOI:** 10.1371/journal.pone.0067261

**Published:** 2013-06-24

**Authors:** Holly E. Copeland, Amy Pocewicz, David E. Naugle, Tim Griffiths, Doug Keinath, Jeffrey Evans, James Platt

**Affiliations:** 1 The Nature Conservancy, Lander, Wyoming, United States of America; 2 Wildlife Biology Program, University of Montana, Missoula, Montana, United States of America; 3 Natural Resources Conservation Service, Bozeman, Montana, United States of America; 4 Wyoming Natural Diversity Database, University of Wyoming, Laramie, Wyoming, United States of America; 5 The Nature Conservancy, Laramie, Wyoming, United States of America; 6 The Nature Conservancy, Minneapolis, Minnesota, United States of America; University of Alberta, Canada

## Abstract

Increasing energy and housing demands are impacting wildlife populations throughout western North America. Greater sage-grouse (*Centrocercus urophasianus*), a species known for its sensitivity to landscape-scale disturbance, inhabits the same low elevation sage-steppe in which much of this development is occurring. Wyoming has committed to maintain sage-grouse populations through conservation easements and policy changes that conserves high bird abundance “core” habitat and encourages development in less sensitive landscapes. In this study, we built new predictive models of oil and gas, wind, and residential development and applied build-out scenarios to simulate future development and measure the efficacy of conservation actions for maintaining sage-grouse populations. Our approach predicts sage-grouse population losses averted through conservation action and quantifies return on investment for different conservation strategies. We estimate that without conservation, sage-grouse populations in Wyoming will decrease under our long-term scenario by 14–29% (95% CI: 4–46%). However, a conservation strategy that includes the “core area” policy and $250 million in targeted easements could reduce these losses to 9–15% (95% CI: 3–32%), cutting anticipated losses by roughly half statewide and nearly two-thirds within sage-grouse core breeding areas. Core area policy is the single most important component, and targeted easements are complementary to the overall strategy. There is considerable uncertainty around the magnitude of our estimates; however, the *relative* benefit of different conservation scenarios remains comparable because potential biases and assumptions are consistently applied regardless of the strategy. There is early evidence based on a 40% reduction in leased hectares inside core areas that Wyoming policy is reducing potential for future fragmentation inside core areas. Our framework using build-out scenarios to anticipate species declines provides estimates that could be used by decision makers to determine if expected population losses warrant ESA listing.

## Introduction

Land use change is rapidly occurring throughout the western United States (US) due in part to both rising energy demand and interest in domestic energy production related to national security concerns. In the Intermountain West, for example, a doubling of oil and gas development occurred between 1990 and 2007 [1]. In addition to fossil fuels, the West has some of the best renewable energy resources, specifically wind and solar, in the US [2]. States with renewable energy mandates are further propelling demand for new renewable power sources. Coupled with energy development, rural areas with desirable natural amenities and recreational opportunities have experienced a surge in rural development since the 1970s [3], with growth in the Intermountain West during the 1990s occurring faster than any other region of the country [4].

Of particular concern is the spatial overlap between energy and residential development and Greater sage-grouse (*Centrocercus urophasianus;* sage-grouse) populations, especially in Wyoming, where 37% of the world’s sage-grouse population resides [5] and where 69% of sage-grouse habitat overlays federal mineral estate, of which 52% has been authorized for exploration and development [1]. Sage-grouse require large and intact sagebrush habitats to maintain viable populations [6]–[9]. When human developments fragment sagebrush habitats, sage-grouse populations are negatively affected either directly or indirectly [8]–[10]. Direct fragmentation impacts typically include loss of breeding or foraging habitat. Indirect impacts can result from changes in habitat quality, predation, noise, or disease [1], [11], [12]. Both types of fragmentation can result in population loss, therefore limiting habitat fragmentation is assumed to be an important strategy for the long-term maintenance of sage-grouse populations (Fish and Wildlife Service 2010).

Sage-grouse are at the epicenter of one of the largest conservation experiments ever undertaken in North America with an unprecedented number of state governments, federal agencies, industry partners and conservation organizations mobilizing to develop successful conservation strategies to proactively reduce the need to list this candidate species [13] under the US Endangered Species Act (ESA). As part of this effort, Wyoming implemented a “core area strategy” on August 1, 2008 (State of Wyoming Executive Order 2008-2) that limits infrastructure development within areas having the highest sage-grouse population densities [13] and the Bureau of Land Management (BLM) followed with a statewide Instructional Memorandum (IM) on February 10, 2012 [14] calling for reduced additional management changes to benefit sage-grouse on public lands. In addition, revisions of BLM Resource Management Plans (RMPs) are underway across sage-grouse range to consider sage-grouse needs.

Concurrently on private lands, $100 million has been applied by land trusts on voluntary conservation easements to reduce development threats. Conservation easements, legal agreements with landowners to restrict development rights on their lands in exchange for tax incentives or cash or both, have become a primary protection tool used by governmental agencies and land trusts globally, though especially in the United States, to achieve conservation goals and permanently restrict development and fragmentation on private lands [15], [16]. Easements have been shown to reduce development and favor wildlife use in sagebrush ecosystems [17]. Easements are also expected to be effective for sage-grouse conservation on private lands, but this assumption has not been tested, nor is it known how many easements and at what cost are needed to make a meaningful contribution to sage-grouse conservation.

Missing from state and federal policy and ESA decision-making is a spatially-explicit evaluation of the adequacy of regulatory mechanisms and efficacy of conservation easements. Modeling threats to species and understanding the relationship between these threats and population declines is paramount to finding the balance between development and species conservation. Inherent to this process is an understanding of where development can occur without threatening species viability and whether the conservation actions taken are enough to prevent further declines.

Here we provide an analysis for those charged with conserving sage-grouse that quantifies the relationship between fragmentation and population decline and applies that relationship to different development growth and conservation scenarios. An earlier study [13] noted the importance of using information on the vulnerability of landscapes to guide conservation strategy development since resources are limited and threats are many. By explicitly defining the relationship between development and declines, we demonstrate how conservation can be targeted to areas with the highest concentration of sage-grouse and greatest likelihood of being impacted by development, in order to provide a high return on conservation investments. Conservation action informed by scientific data on strategy effectiveness is more credible and hence more likely to be implemented [18].

**Figure 1:**
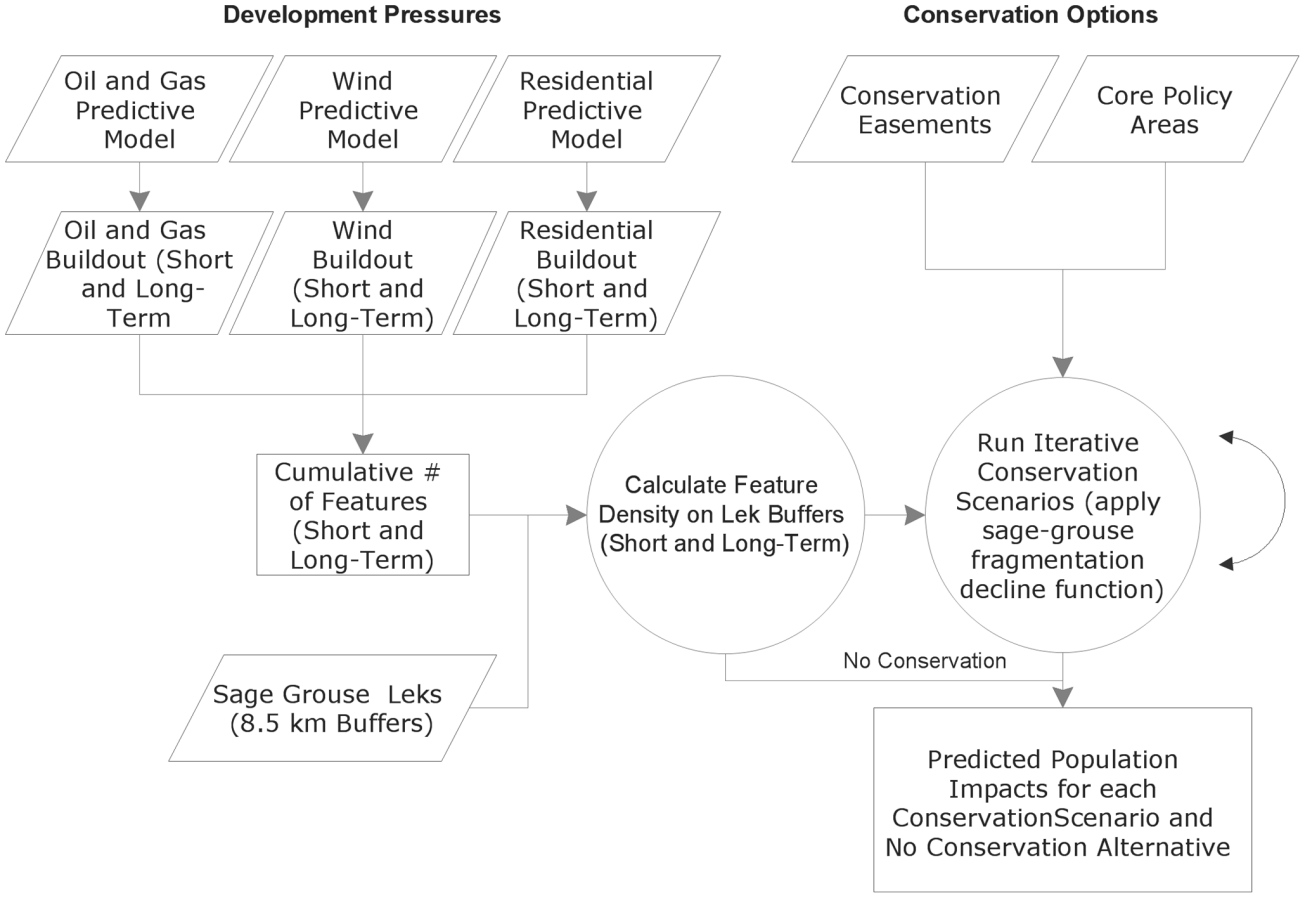
Conceptual model of project and data workflow. Conservation easements are randomly selected from private parcel data, a process that is incorporated into the iterative scenarios script.

This analysis builds on our previous work that measured the impacts of projected oil and gas development on sage-grouse populations throughout their eastern range [19]; however, this first effort did not quantify how proposed conservation action might abate sage-grouse population loss and did not include future impacts from wind or residential development. In this paper, we built new models of future oil and gas, wind, and residential development, and test the success of these models at predicting development. We used these models to evaluate the effectiveness of conservation easements and Wyoming’s core area policy at conserving sage-grouse populations and developed multiple future scenarios to measure the costs of easements relative to the numbers of birds protected. We evaluated expected population loss and male strutting location (lek) extirpations statewide and within core areas (Figure 1).

Our research addressed the following questions: (1) Can our models reliably predict future oil and gas, wind and residential development? (2) How much sage-grouse population loss can be averted by conservation easements and the sage-grouse core area policy? (3) Where are the greatest population losses expected, and how can conservation be targeted spatially to maximally benefit sage-grouse? And lastly, (4) Did the amount of land leased for oil and gas exploration and development decline inside core areas following policy enactment? Such a decline would provide early evidence that industry activities are being modified to make this strategy viable and that Wyoming policy is changing the course of future fragmentation inside core areas.

## Methods

**Figure 2:**
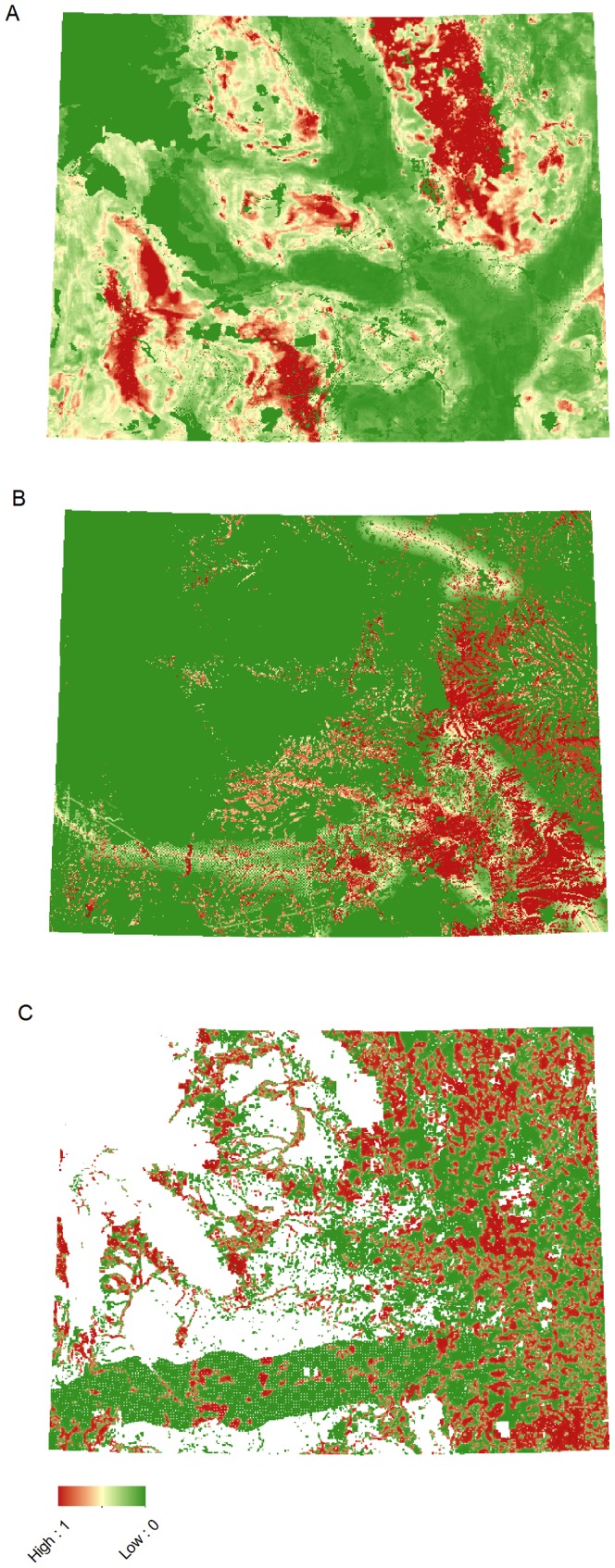
Predictive surface models showing potential for development (0 = low to 1 = high). Figure 2A shows the oil and gas model. Figure 2B shows the wind development model. Figure 2C shows the residential development model.

To predict the vulnerability of sage-grouse to future fragmentation, we modeled cumulative future fragmentation anticipated across Wyoming as a result of projected growth in residential, wind, and oil and gas development and related the modeled fragmentation to sage-grouse population impacts under two scenarios representing short and long-term growth in development (Figure 2). The time frame representing short and long-term development varies by model. Short-term represents expected development within 15–20 years. Long-term represents anticipated development varying from a doubling of expected wind and residential development, to maximum build-out of the oil and gas model, and therefore does not apply to a specific time window. We built raster-based models of development potential and used existing growth projections to populate the landscape with features (houses, wind turbines, oil and gas wells), guided by the predictive models. Our scenarios varied by two funding levels for conservation easement protections ($100 and $250 million (randomly placed within core areas and targeted) and were implemented with and without Wyoming’s core area policy in place.

### Forecasting and Building out Oil and Gas Development

We mapped oil and gas development potential using methods developed for a previous study [19] wherein Random Forests was used to develop a new model of energy potential. Our binary response variable was geospatial data representing producing and non-producing oil and gas wells [20]. Nine topographic, geological and geophysical variables were used to predict development, including data on bedrock geology and Euclidean distance from geologic faults both from the Wyoming State Geological Survey [21]. We used a nonparametric ‘Random Forests’ model in R [22], developed to address statistical issues related to over-fit and parameter sensitivity in CART (Classification and Regression Tree) models [23]. We produced both binary and continuous models of oil and gas development potential and modified the Random Forests generated models with spatial data on legal constraints such as federally protected national parks and wilderness that could prevent development in some locations.

Model validation was performed using out-of-bag (OOB) testing techniques to produce standard error statistics including area under the receiver operating characteristic curve (ROC AUC) [24], Cohen’s kappa [25], OOB error, and class error. Within the Random Forest models independent boot-strapping (OOB) subsets with many thousands of iterations are used for model validation and each tree is constructed using a different bootstrap sample from the original data. This method has been shown to be unbiased in many tests and a reliable indicator of error [23], [26]. The ROC AUC was 0.83, Cohen’s kappa was 0.62, OOB error was 22.4%, and overall model accuracy (total number of correct classifications divided by the total number of sample points) was 82.5%.

**Figure 3:**
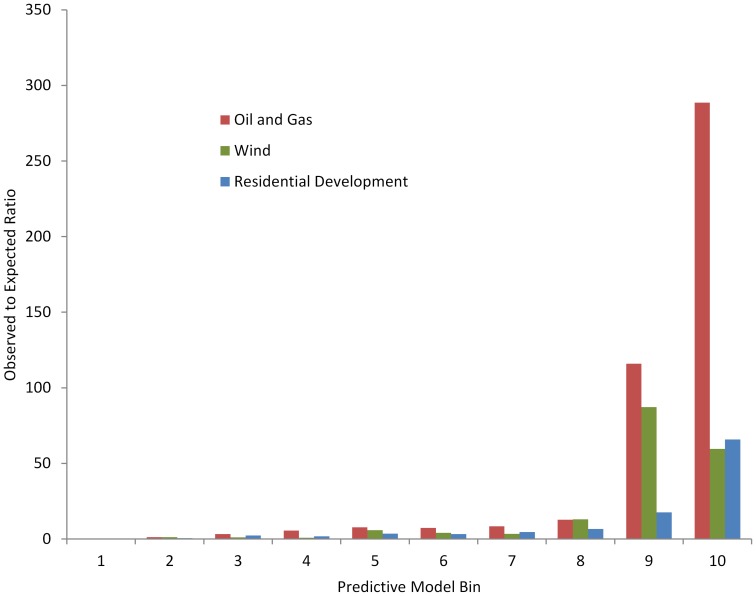
Boyce Index validation of the predictive development models. The Boyce Index uses a Spearman rank correlation to test the area-adjusted frequency of observed to expected validation points falling within a bin and the associated bin’s rank. This graph plots the ratio of observed to expected points of independent test data for each model against the model bins (higher bin = higher likelihood).

Additionally, we applied the Boyce Index to measure observed versus expected occurrence, using independent validation data points and binned versions of the models. The Boyce Index uses a Spearman rank correlation to test the area-adjusted frequency of validation points falling within a bin and the associated bin’s rank. To test the oil and gas model, we used producing wells between 2008 and 2012 (N = 6,240) as the independent dataset. We partitioned the model predictive data into 10 ordinal area-adjusted classes (bins) and for each class *i*, we calculated two frequencies: 1) P_i_, the predicted frequency of evaluation points and 2) E*i*, the expected frequency of evaluation points. We applied a Spearman-rank correlation to test *predicted-to-expected* (P/E) ratio F*_i_* given by:
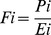



If the model accurately predicts oil and gas development, lower bins should have fewer producing wells than expected by chance, resulting in F*_i_* <1 and conversely higher bins should have F*_i_* increasingly greater than 1 indicating more wells than expected by chance. Our results were consistent with a highly significant model (Figure 3) (Boyce Index = 0.99; P<0.001) [27].

As a final validation test, we examined the error distribution, and found that the median absolute deviation (MAD) from the median error variance, where errors converge in the model, was small (0.004). Thus, all validation statistics indicated a stable and acceptable model. Additional model details are provided in Text S1.

We populated a build-out model under four alternative future scenarios: short-term and long-term (with and without the core area strategy). Projections for future wells were obtained from Bureau of Land Management Resource Management Plans [19]. The “short-term” scenario used the continuous model of oil and gas development to place 52,626 wells on the landscape first in cells with the highest likelihood values and filled those cells to the maximum allowable density before moving to the next cells. The “long-term” scenario used the binary model to place 155,706 wells in every cell with a positive binary value at the highest allowable density. The core area strategy scenarios restricted development to one well per section (259 hectares) within core areas [28]. For the short-term scenario, the total number of wells did not change with the core area policy, but was reduced by 20% (31,036 wells) for the long-term scenario because all suitable areas had already been developed outside core areas.

### Forecasting and Building out Wind Energy Development

We forecasted wind energy development potential through a two-step modeling process that included a predictive model to represent wind resource potential, followed by adjustments to reflect expected short-term development and legal or operational constraints. We fit the predictive model using maximum entropy methods, a machine learning technique that models geographic distributions using presence-only records [29], [30], using Maxent® software version 3.3.3e. The response variable was existing wind turbines [31] and predictor variables were the average wind resource potential at 50-m height [32], percent slope, and topographic position (150 cell neighborhood, canyon threshold −10, ridge top threshold 10, slope threshold 6) [33]. To validate the modeling approach, we fit a hind casting model where turbines from wind farms constructed until 2008 were used as training data (502 turbines, 17 farms) and turbines from wind farms constructed post-2008 were test data (460 turbines, 11 farms). The hind casting model validation performed well, with a test AUC of 0.864.

The Maxent® model represented the quality of wind resources but did not prioritize where development would most likely occur in the near term. Therefore, we adjusted the model using short-term development indicators, including density of existing meteorological towers, distance to proposed transmission lines, proposed wind farm boundaries and land tenure. We excluded locations where development was precluded due to legal or operational constraints, including protected lands (e.g. wilderness areas, conservation easements), airport runway space, urban areas, mountainous areas above 2743-m, and open water. We tested the adjusted model with the Boyce Index [27] using methods similar to testing the oil and gas model as described above with turbines constructed in Wyoming between 2009–2010, which indicated a highly significant model (Boyce Index = 0.89; P = 0.001). Additional model details are provided in Text S1.

We built out wind development using the modeled probabilistic surface to guide where new wind turbines were placed. For the short-term scenario projecting wind development in 2030, we used existing 20 year projections for Wyoming (11.42 GW, [34] resulting in 4569 new turbines (assuming 2.5 MW per turbine). For the long-term scenario (2050, at the current projected rate), we doubled the number of new turbines to 9138. To build out these turbines, we randomly selected one of the raster cells with the highest wind development potential and randomly placed an initial turbine in the northern half of that cell. Additional turbines were successively placed 300 m south of the initial turbine until the cell boundary or limit of 3 turbines per 1-km cell was reached. This spacing reflects the typical distance between 2.5-MW turbines. To simulate the clustering of turbines within wind farms and their typical north-south orientation, we increased the wind potential of the cells immediately north and south of the cell that was just built upon by 0.05, an adjustment factor that best approximated typical wind farm size. To implement the core area policy, wind turbines were excluded from core areas [28]; the total number of turbines remained unchanged.

### Forecasting and Building out Residential Development

To predict where new housing units were most likely to be built, we modeled the change in housing unit density over the past 20 years (1990–2010, US Census data) within R [22], using Random Forests and spatially-explicit predictor variables previously related to residential development patterns in Wyoming, including services, transportation, natural amenities, and past residential and oil and gas development [35] (Table S1). A binary model was selected as the best-fitting model and was used to predict a probabilistic output [36]. Cohen’s kappa was 0.75, OOB error was 11.7% and model accuracy was 84.9% and 90.5%, respectively, for observed positives (change) or observed negatives (no change) correctly predicted. We also used the Boyce Index [27] to test the model against residential structures constructed in Wyoming between 2010–2012 [37],which indicated a highly significant model (Boyce Index = 0.976; P<0.0001). Additional model details are described in Text S1.

We built out residential development using the modeled probabilistic surface to guide placement of new housing structures. We first populated the private lands parcel database with the mean likelihood of development from the modeled surface. Next, we excluded parcels from development that had an existing conservation easement or other special land protection, if they were located within incorporated cities, or if their area was less than 2 hectares and they were located within 8 km of a city. We assumed that most small parcels close to cities were already developed. For eligible private parcels occurring within the highest three quantiles of mean development probability, we randomly placed new housing units at least 10 meters apart until the housing projections were met for each county.

For each county we determined two levels of projected rural housing development (Text S1), using the Wyoming housing forecast’s “moderate” (short-term) and “very strong” (long-term) projections, which are based on expected changes between 2010 and 2030 [38]. The expected rate of development for each county was determined from the housing forecast projections and applied to 2010 census data reflecting actual household numbers to determine the number of future households expected per county. Since we were interested primarily in rural development, we determined the proportion of new households expected in the incorporated cities of each county [38] and subtracted these from county totals to determine expected new rural households (Table S2). Finally, we converted occupied households to housing units/structures using 2010 census county-level ratios of occupied to unoccupied housing units. Residential development is not restricted by the core area policy, so this build-out remained unchanged with and without the core area policy.

### Parcel Valuation

We acquired geospatial parcel data from each county assessor’s office of all parcels. Parcels smaller than 50 acres were removed from the database, and remaining parcels classified as agricultural were separated into two categories, grazed or irrigated, using a spatial dataset of irrigated lands in Wyoming [39]. We estimated fair market parcel value using data from a report valuing types of agricultural lands in Wyoming by region and multiplied the appropriate regional values by parcel acres [40] (WY Grazing Land and WY Irrigated Meadow for grazing and irrigated parcels respectively). We estimated the easement cost at 50% of the calculated parcel value, which approximates typical easement valuation for Farm Bill programs in Wyoming (personal communication Paul Shelton).

### Sage-grouse Fragmentation Decline Function

We applied a generalized linear regression model to quantify the relationship between sage-grouse male lek population abundance and feature density (feature density = the number of wells and wind turbines per square kilometer within an 8.5 km circular buffer). No such model existed for our entire study area at the appropriate scale for our modeling, and that included wind turbines. We were unable to include residential development, because accurate housing locations are not available in rural areas of Wyoming. We assumed that residential development has similar impacts as oil and gas and wind development to sage-grouse based on research finding a strong negative relationship between lek count trends and proportion of the landscape developed (classified as urban, suburban, or a highway) within 5 km or 18 km ([41]).

Sage-grouse lek data were acquired from the Wyoming Game and Fish sage-grouse lek location and status database (downloaded January 20, 2012). We analyzed 1347 active leks, using the peak male count from 2009–2011 because not all leks are counted each year, but most are counted within a three-year interval. We choose to use the peak male count for the last three years because development of the Wyoming core area policy was based on this method [13]. The response variable was peak male lek attendance from 2009 to 2011. High site fidelity but low survival of adult sage-grouse, combined with lek avoidance by younger birds [42], has resulted in a time lag between development and lek loss. We assumed a four year time lag based on two studies demonstrating a 3 to 4 year lag in population response to development in Montana and Wyoming [8], [43], and research since then supports time lags that vary between 2–10 years [44]; We calculated the density of wind turbines and oil and gas wells within an 8.5 km buffer around each lek to capture the majority of the nesting and breeding population based on findings that 80% of nests are distributed within that distance [7].

**Figure 4:**
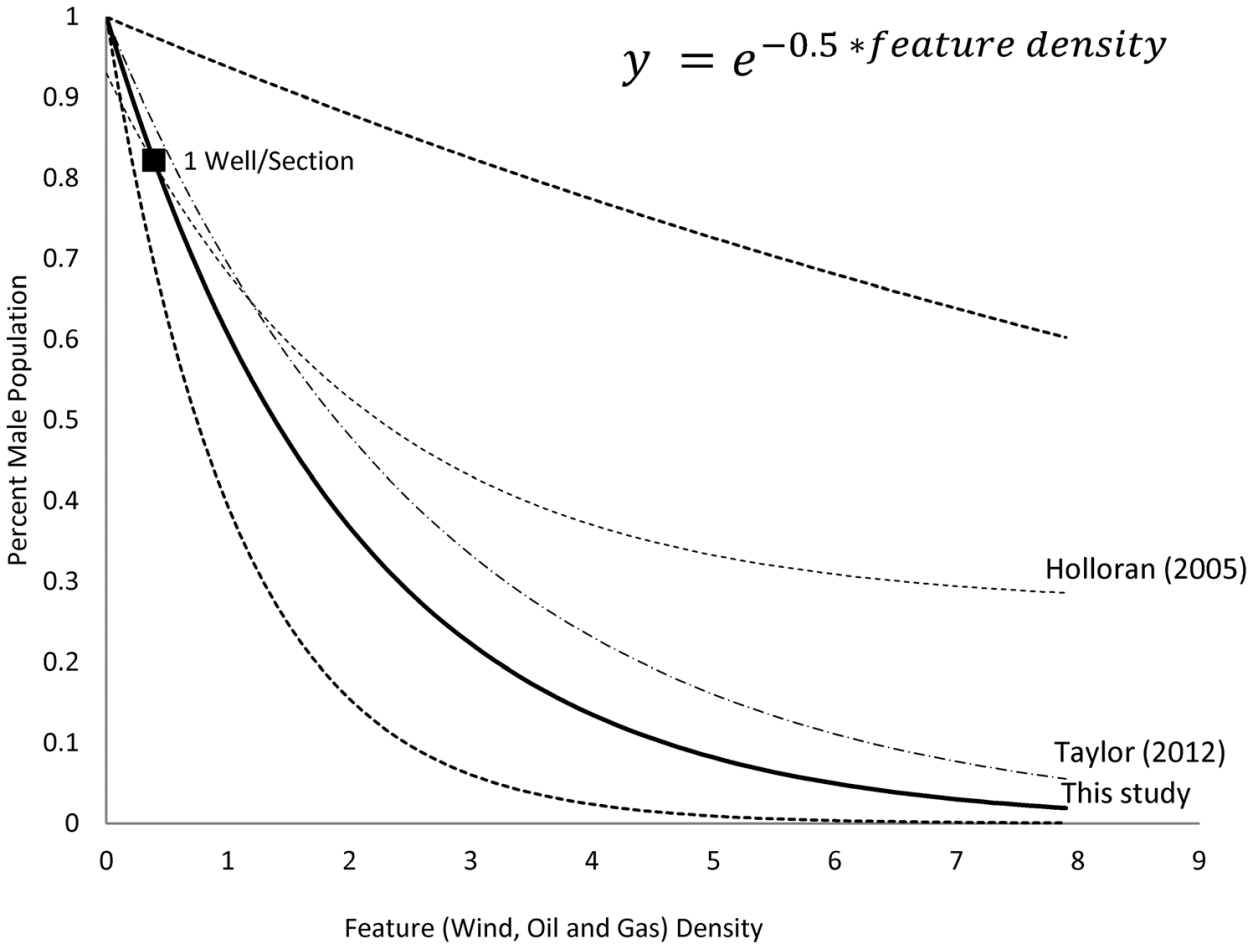
Sage-grouse population response to feature density regression relationship (solid line) with 95% CI (dotted lines). The feature density at one well per section is shown for reference, as well as the regression relationships for two similar studies.

The sage-grouse lek data includes a large number of zero abundance leks. To account for these zeros and normalize the dataset, we generated feature class density quantile classes, and ran a linear regression on the log of the mean population abundance on feature density classes (*p = 0.035, R^2^ = .82, coefficient 95% CI* −*0.934,* −*0.064*) and yielded the following sage-grouse fragmentation decline function, based on the model coefficient: *population abundance* = *e* ^(-.5 * feature density)^ (Figure 4).

We are aware of other recently published linear and non-linear regression models of lek attendance and well density [43]–[46]. We considered various regression models to fit these data based on two published studies and one unpublished report; however, we were restricted in model selection to one with interpretable coefficients that could be applied broadly within our complex modeling framework. Nonetheless, we compared our model fit to other similar models [43], [46] and found ours to be consistent with this literature, more conservative, and that our 95% confidence intervals comfortably bounded the other models (Figure 4).

### Conservation Scenarios

Sixteen scenarios were developed to represent $100 million (random) and $250 million (random and targeted) in conservation easement purchases with and without the core area policy in place, statewide and with core area leks only. Amounts were based on 2011 Farm Bill funding levels in Wyoming ($100 million including required partner matching funds), and a possible future funding level. We simulated the efficacy of the core area policy by altering the future density of energy development, as described previously, in accordance with the policy. Each privately owned parcel, as described in the parcel valuation section, was assigned a number of projected development features. Parcels with existing easements or other protections were not assigned projected features.

**Figure 5:**
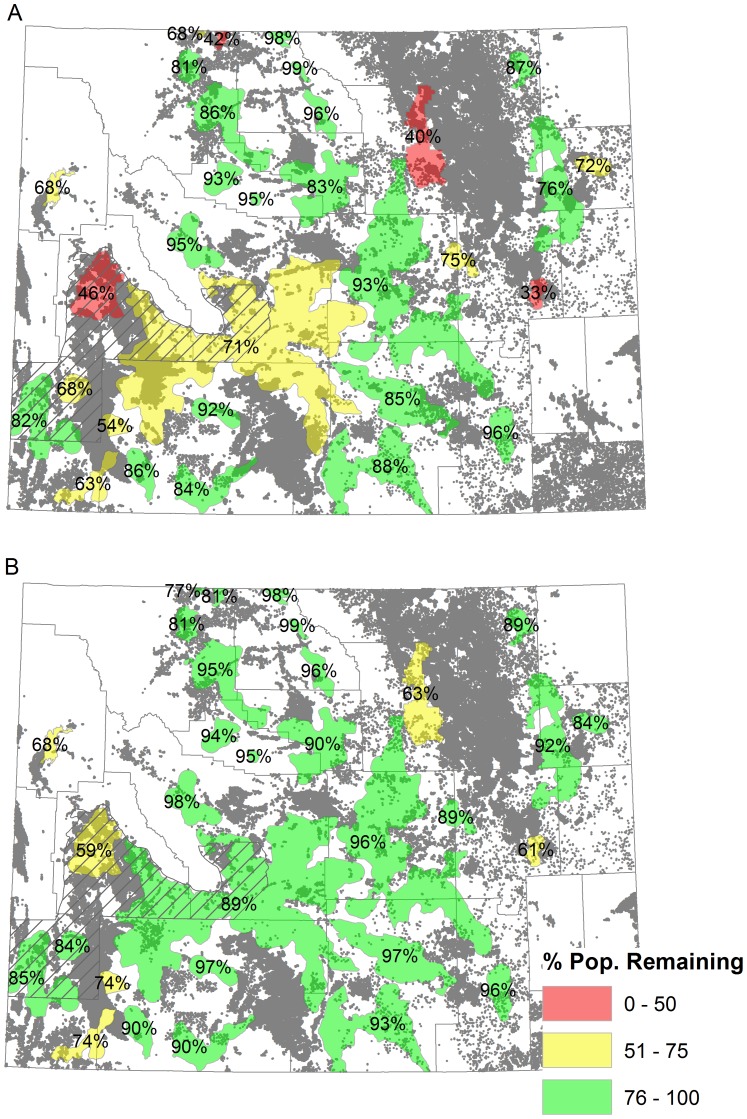
The percent of the sage-grouse population remaining under the long-term scenario with and without conservation. Figure 5A represents the population remaining from current in each core area and the build-out features shown as gray dots under the long-term scenario with no conservation. Figure 5B shows the percent of the sage-grouse population remaining from current in each core area and the build-out features shown as gray dots under the long-term scenario with the core area strategy in place. The NRCS easement priority area is shown in hatched shading, and county outlines in gray.

For the random scenarios, we used a parcel-based stochastic model programmed with Python for ArcGIS [47] to simulate the purchase of new conservation easements within core areas [48] with 50 iterations per scenario (variation in output metrics stabilized at 50 iterations) until the total scenario funding amount was reached. We applied additional purchase targeting in our model to reflect a 2011 Natural Resources Conservation Service (NRCS) effort that identified an easement purchase priority area in southwest Wyoming (shown on Figure 5a) to target easements in areas with the largest and densest sage-grouse populations. Therefore, our model randomly selected 50% of easements from the southwest priority area and 50% of easements from the remainder of the state.

For the targeted $250 million dollar scenarios, we applied a population weighted kernel density function (radius = 15km, cellsize = 1km) to all lek centers with an expected population less than 75% of the current population under the long-term scenario. We sorted parcels from highest to lowest risk and selected parcels where sage-grouse populations are predicted to decline below 75% of the current population levels under the long-term scenario until $250 million in easement value had accumulated. This process was repeated for parcels intersecting core areas only.

Once easements were selected in a scenario, feature density was recalculated within each 8.5 km lek buffer based on the assumption that the easement would remove future development potential. Future development on selected parcels was assumed to be averted; a key assumption that may not hold true under the split-estate parcels where the surface landowner does not hold the mineral rights. In practice, however, per NRCS policy, a minerals review is conducted prior to easement selection and parcels with high development potential are avoided or severe restrictions are placed on well development. Using the sage-grouse fragmentation decline function, we then recalculated based on the new feature density, our resulting prediction for the number of sage-grouse at each lek under each scenario. The number of sage-grouse projected at each lek was summed to report the predicted number of sage-grouse across all leks in the scenario (for the random scenarios we calculated the mean number of sage-grouse predicted across all 50 model runs).

We evaluated efficacy of the core area policy alone under the long-term scenario by calculating expected population loss within core areas with and without the core area policy. Similar to easement scenarios, cumulative feature density was calculated with and without the core area policy and the sage-grouse fragmentation decline function applied, resulting in the projected number of sage-grouse males at each lek. The number of males at leks within all core areas was summed and divided by the current male population to yield the percent of the population remaining with and without the core area policy.

To understand the spatial configuration of potential lek extirpations, we mapped leks under the long-term scenario modeled to be extirpated with and without the core area policy in place. We defined a lek as extirpated if the long-term scenario predicted 0 or 1 males at the lek.

### Assessing Model Uncertainty and Sensitivity

Interpretation of outcomes is related to strength of inference, yet estimating the inherent uncertainty in any spatial modeling analysis is difficult and appropriate techniques are limited. Uncertainty in this study arises from sources of error introduced by model structure, input from spatial datasets, and imperfect lek-based count data. We account for uncertainties by applying appropriate validation or uncertainty measurement techniques for each modeling step. First, we validated each predictive model (oil and gas, wind, and residential) in using multiple methods appropriate to the each type of model, and we also validated each model with independent datasets using methods developed for testing resource selection function (RSF) models (Figure 3) [27]. Second, we ran stochastic simulations for our build-out models using multiple iterations until the variance stabilized. Third, we used short and long-term growth scenarios to span the broad range of possible outcomes. Finally, we tested the sensitivity of our final output to variation in the response of sage-grouse to development by propagating 95% confidence intervals from the regression through our model to provide final results bounded by these estimates.

### Oil and Gas Leasing Activity Inside Core Areas

The BLM Wyoming State Office Reservoir Management Group provided a tabular summary of acres of federal oil and gas leases within core areas from June 1, 2006 through January 1, 2013. Lease area was separated by active unproven leases and leases held by production within core areas and reported for every four month time period.

## Results

Our analysis predicts a 14% (95% CI: 4–30%) (short-term) to 29% (95% CI: 8–46%) (long-term) statewide sage-grouse population decline and 11% (95% CI: 3–25%) (short-term) to 24% (95% CI: 6–40%) (long-term) within core areas from cumulative land use change *in the absence* of conservation measures that prevent fragmentation (Table 1). We estimate that the core area strategy alone reduces these declines statewide to 9–15% (95% CI: 3–32%) and 6–9% (95% CI: 2–24%) within core areas.

**Table 1:** Projected core area sage-grouse population declines under short- and long-term development scenarios, with and without conservation actions.

Scenario	Population Decline Predicted in Core Areas in Number of Males (% of Current Core Population)	Sensitivity Test (%)	Population Decline Predicted Statewide in Number of Males (% of Statewide Population)	Sensitivity Test (%)
Short-term, no conservation	3,421	1,021–8,034	5,385	1,659–11,772
	11%	3–25%	14%	4–30%
Short-term, core area policy	2,263	802–6,902	4,229	1,438–10,707
	7%	3–21%	11%	4–27%
Short-term, core area policy, $100 M random easements	2,189	790–6,825	4,164	1,426–10,633
	7%	2–21%	11%	4–27%
Short-term, core area policy, $250 M random easements	2,086	774–6,714	4,073	1,409–10,531
	7%	2–21%	10%	4–27%
Short-term, core area policy, $250 M targeted easements	1,807	732–6,461	3,674	1,342–10,208
	6%	2–20%	9%	3–26%
				
Long-term, no conservation	7,705	1,913–13,018	11,212	2,937–18,028
	24%	6–40%	29%	8–46%
Long-term, core area policy	3,802	1,048–8,706	7,037	1,986–13,542
	12%	3–27%	18%	5–34%
Long-term, core area policy, $100 M random easements	3,676	1,026–8,572	6,919	1,965–13,410
	11%	3–27%	18%	5–34%
Long-term, core area policy, $250 M random easements	3,498	995–8,392	6,767	1,938–13,247
	11%	3–26%	17%	5–34%
Long-term, core area policy, $250 M targeted easements	2,948	898–7,865	6,066	1,812–12,685
	9%	3–24%	15%	5–32%

**Figure 6:**
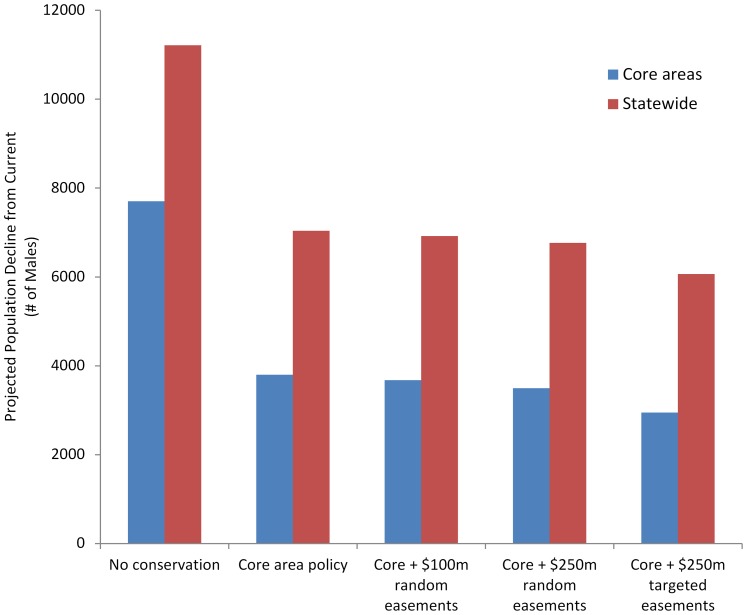
Total predicted male population declines in long-term growth scenarios compared to the no conservation scenario.

When we simulated the *random* acquisition of conservation easements at $100 million and $250 million dollar levels in addition to the core area policy, these actions had a small effect and only reduced anticipated population declines by 1% or less, whereas $250 million in targeted easements reduced declines by up to 3% (Table 1). The core area policy with the addition of $250 million in *targeted* easements was the most effective conservation strategy and averted expected population declines in the long-term scenario by up to 46% statewide and 62% within core areas (Figure 6). In this most effective scenario, targeted easements averted an additional 9–11% of expected declines compared to that of the core area policy alone.

**Figure 7:**
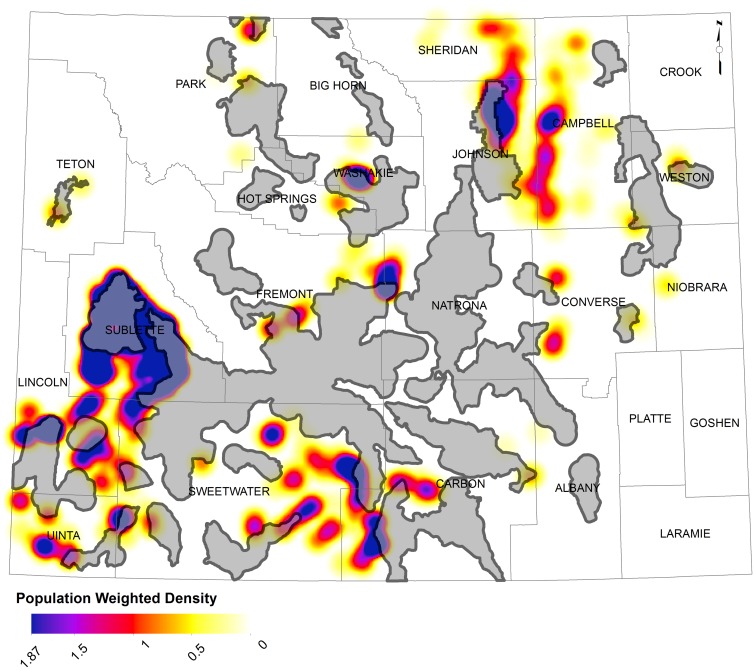
Population weighted kernel density map of leks with a predicted decline less than 75% of the current population under the long-term scenario (yellow = lower decline, blue = higher decline. The core policy areas are shown in gray.

Our analysis of populations within core areas shows that the benefits of the core area policy varies spatially and are concentrated in southwest and northeast Wyoming where private lands and high residential growth co-occur (Figure 5, Figure 7). Under long-term scenarios, the number of core areas predicted to maintain >75% of their current populations increases from 20 without conservation to 25 with the core area policy (out of 31), and all core areas move above the 50% threshold (Figure 5). In absolute numbers, we predict that maintaining core policy long-term will avert the loss of more than 4,175 (95% CI: 951–4,486) males statewide.

**Figure 8:**
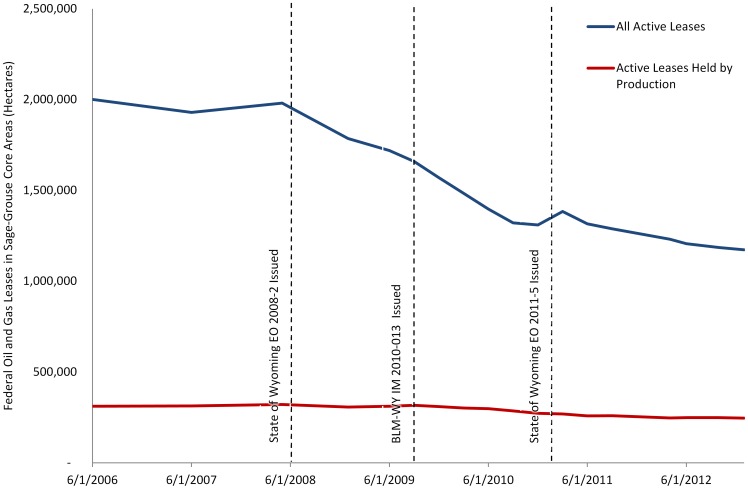
Federal oil and gas leases in Wyoming within sage-grouse core areas (Wyoming Governor’s Version 3). Total hectares of active leases are shown in blue and hectares of active leases held by production are shown in red.

The BLM data shows a 40% reduction in area leased for oil and gas exploration and production inside core areas since August 1, 2008 when the core area policy was first enacted (1,980,849 hectares May 2008 versus 1,172,735 in January 2013; Figure 8). Similarly, leased area held by production declined by 23% during the same time period (320,816 hectares versus 246,514). The observed leasing patterns are attributable to expirations, terminations, parcel deferrals, and some additional leasing in accordance with the sage-grouse IM [14], resulting in a decline in the total amount of hectares leased in core areas.

**Figure 9:**
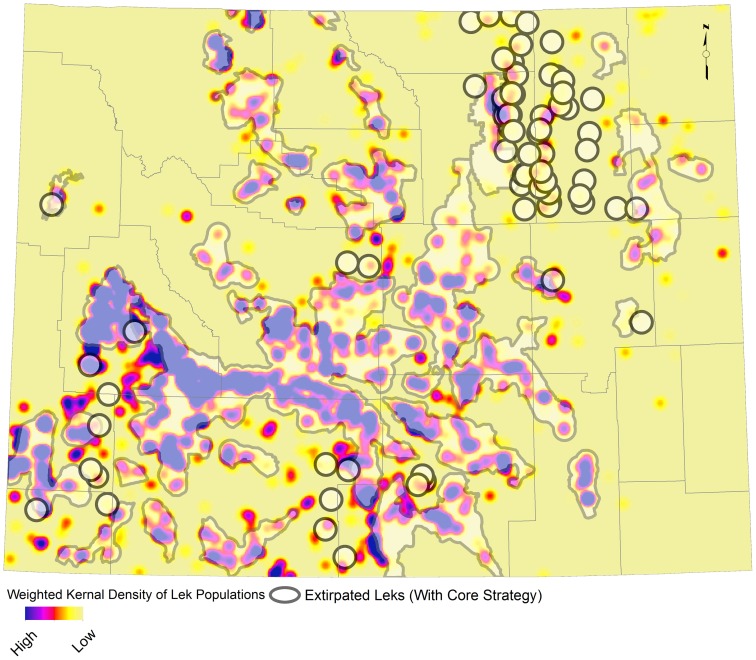
Population weighted kernel density map of all leks. Circles represent leks extirpated under the core area policy. Wyoming designated core areas are shown in gray and county boundaries are shown for reference.

We examined which leks statewide are likely to be extirpated with and without the core area policy. We predict a total of 98 (7%) leks extirpated without the core area policy, and 75 (6%) leks extirpated with the core area policy in place (Figure 9). No leks located within core areas are predicted to be extirpated with the core policy in place. Extirpated leks are concentrated outside core areas in the Powder River Basin where more than 30,000 oil and natural gas wells had already been drilled prior to core area policy enactment.

## Discussion

Sage-grouse have fared poorly as its western US range has been highly altered and modified by development and land cover change over the past century. These changes have resulted in regional sage-grouse population declines ranging from 17–47% [49]. Unchecked by conservation, expected development in the long-term could threaten an additional 29% (95% CI: 8–46%) of the remaining population in Wyoming. Comparatively, our analysis predicts that the core area policy plus a targeted $250 million easement investment could reduce anticipated population losses to 9–15% (95% CI: 3–32%), cutting anticipated losses by roughly half statewide and nearly two-thirds within core areas. Core area policy is the single most important component, and targeted easements are complementary to the overall strategy, averting an additional 9–11% of expected declines. Our approach explicitly models averted declines and therefore only population loss; however, management practices could be implemented either within core areas or elsewhere range-wide that could result in population gains and offset these losses.

Targeting easements to areas with a high threat of residential subdivision and dense sage-grouse populations is critical as our analysis suggests that random placement of easements within core areas has a much lower potential for benefiting sage-grouse populations. We found that the biological benefits of easements are concentrated primarily in southwest Wyoming; conservation practitioners can maximize return on investment by targeting conservation efforts with willing landowners on private lands having high bird abundance and subdivision risk [18] (Figure 7). This finding may be especially relevant to federal and state agencies that use public funds to pay for easements, are sensitive to costs, and typically seek the highest return on investment. The core area policy and targeted conservation easements together provide a unified approach that could effectively contribute to conserving the diverse seasonal habitat needs required to maintain sage-grouse populations. Comingled land ownership in the West is typified by publicly-held breeding and nesting habitats in dry uplands and privately-owned mesic riparian habitats in productive lowlands. Policy addresses fragmentation from energy development on most public and private lands within core areas, while conservation easements reduce the residual threat of subdivision on private lands. Moreover, highly productive riparian habitats characteristic of private lands are critical to chick survival, a vital rate known to drive sage-grouse population growth [46].

We have attempted to quantify the most important factors influencing sage-grouse conservation success. As is the case in complex modeling environments, there are many factors that we were not able to consider and assumptions made that may over- or underestimate conservation outcomes. We used lek count data as a surrogate for overall population health and believe that it provides a reasonable index to relative abundance, but counts are subject to observer bias and all lek locations are not known [50]. We did not consider some factors known to affect sage-grouse viability, including other types of infrastructure development (i.e. mining, roads, transmission lines) [51], exotic plant species invasions [41], and associated increased predation [52] and West Nile virus [46]–all of which may underestimate declines. West Nile is a threat to sage-grouse in low to mid elevation populations [53], but focusing easement investments in southwest Wyoming is preferred because the risk of West Nile virus is low, even under altered climate scenarios [11]. The 8.5-km scale at which lek data were analyzed could bias predictions either way. Core area delineations are assumed to include all seasonal habitats for grouse but anticipated benefits may be biased high if birds use areas beyond the buffered leks when migrating between seasonal habitats, for brood-rearing, and as winter habitat. Alternately, anticipated benefits may be biased low if impacts from human developments dissipate with distance from lek because we did not attempt to model a decay function [46].

We have quantified uncertainty and increased predictive capacity using well-tested, independently validated development models, stochastically simulated build-out scenarios, and sensitivity tests (Figures 2 & 3) [54]. We conducted a sensitivity analysis to the regression to demonstrate how our results would change if sage-grouse responded to development to a greater or lesser degree. While we report the sensitivity data as determined by 95% confidence intervals from the regression, it is imperative to note that these estimates should *not* be considered independent errors. In other words, the *relative* benefit of different conservation scenarios remains comparable because potential biases and assumptions are consistently applied regardless of the strategy and if, for example, bias was high in a given scenario, it should be high for every scenario. That said, the cumulative effect of all sources of uncertainty and bias cannot be calculated and is therefore unknown.

To preempt an ESA listing, the Wyoming Governor’s Office took the unprecedented (for species conservation) step in 2008 of enacting the core area policy. Considerable effort from a diversity of stakeholders has followed to support this policy, including the creation of a “Sage-Grouse Implementation Team” (SGIT) to interpret and set guidelines for the policy. Success of the policy at protecting sage-grouse likely rests, in part, with this team and how it guides uranium, coal, transmission lines or other types of disturbance not well-defined in the plan. Some fear that this lack of specificity has created loopholes in the policy that will render it ineffective. If the integrity of the core area policy is not maintained, the risk of an ESA listing increases at the peril of subsequent development. Our analysis could not and does not address or anticipate these factors. We presume that development is restricted to one feature per section where predicted, and that other additional sources of fragmentation will not occur. Our assumption is not a perfect solution, given that development will certainly be more clumped or dispersed throughout the landscape. Nonetheless, for the purposes of this study, our reported point estimates are accurate plus or minus a factor of two or three, which is sufficient for strategic decision making for which this study is intended.

“Investing” in sage-grouse is a social decision that must include consideration not only the benefit of conservation strategies presented here, but the long-term value of sage-grouse to society and ancillary benefits provided by restoration and protection of sagebrush for its “services” to ranchers, hunters, wildlife enthusiasts, and recreationists. It must also include consideration of the opportunity cost of foregoing development of core areas in exchange for protecting sage-grouse and unfragmented sagebrush habitat [55]. Ultimately, society must decide whether intact sagebrush (and the species inhabiting it) has a higher value long-term value than the short-term gains of revenue from energy development.

As the current arbiter for “society” on endangered species issues in the United States, the US Fish and Wildlife Service alone has the difficult decision of determining if current protections go far enough to conserve a candidate species. There is early evidence based on a 40% reduction in leased hectares inside core areas that Wyoming policy is changing the course of future fragmentation inside core areas (Figure 8) and our study both supports the conclusion that these changes will avert lek decline and provides an estimate of losses averted. Our framework provides some of the data required to determine if current and future risk of population declines warrant ESA protection, given investment in policies and conservation easements to benefit sage-grouse.

Economic ramifications of listing species under ESA are substantial [56] and sage-grouse is no exception. Wyoming has 35.1 TCF of proven natural gas reserves and 567 million barrels of proven oil reserves [57]–clearly billions of dollars and resources are at stake. It is in the interest of all extractive industries operating within sage-grouse range to take a highly proactively approach to secure the future of sage-grouse and the freedom to operate by ensuring the intent of the core area policy is upheld, not undermined by possible loopholes, and everyone is operating under the same rule book. To date, there has been an unprecedented level of public and private spending for sage-grouse with approximately $347 million invested conservation management and protection range-wide. Clearly more will be required for protection and restoration, but these costs must be compared to the cost of litigation or the opportunity cost of foregoing development. The past 15 years of science research for sage-grouse has been largely focused on documenting impacts. We are entering a new era that will require a shift of sage-grouse scientists to measure the benefits of enacted policies and conservation actions and report if these actions are working. Our study is one such effort, but additional studies are needed that complement and build on our approach.

Our approach provides a novel framework for linking predictive modeling with build-out scenarios to anticipate species declines before expected land use changes occur. Our intent is not to suggest that the conservation strategies presented here provide the only solution to reducing threats, but rather to measure their potential contribution to sage-grouse conservation. Other states within sage-grouse range are drafting conservation solutions similar to Wyoming, and efforts to conserve the lesser prairie chickens are embarking on a similar trajectory. The level of conservation effort for sage-grouse is in sharp contrast to the case of caribou in Alberta, also threatened by energy development, where sweeping policies and conservation measures have not been undertaken despite mounting threats from energy development [58]. If successful conservation strategies for sage-grouse can be measured and quantified, these ideas represent an emerging conservation strategy that could be replicated and applied to other imperiled landscape-scale species. Finally, the conservation actions for sage-grouse are suspected to have additional ancillary protections for many other species occupying the same range, such as mule deer and sagebrush obligate songbirds. If scientists can quantify these benefits as well, it will demonstrate that investing in sage-grouse leverages a much bigger win for conservation than is perhaps currently understood, and this realization could help safeguard these protections long into the future.

## Supporting Information

Table S1 **Predictor variables included in Random Forests models of change in housing density.** An asterisk indicates that the variable was included in the selected best-fitting model.
(DOCX)

Table S2 **Projected number of new rural housing structures for each county for short-term and long-term scenarios and incorporated towns from which projected housing structures were excluded.**
(DOCX)

Text S1 **Additional methods text on oil and gas, wind and residential development modeling.**
(DOCX)
