# Supplementary material for: Measuring the Effectiveness of Conservation: A Novel Framework to Quantify the Benefits of Sage-Grouse Conservation Policy and Easements in Wyoming
Source: PLoS One. 2013 Jun 24;8(6):e67261. doi: 10.1371/journal.pone.0067261 (PMC3691158; doi:10.1371/journal.pone.0067261)
Supplement: Table S1 — Predictor variables included in Random Forests models of change in housing density. An asterisk indicates that the variable was included in the selected best-fitting model. (DOCX) [file pone.0067261.s001.docx]

**Table S1**

| **Model predictor variables** | **Data source** |
| --- | --- |
| Services |  |
| Distance to nearest city with  population >50,000 * | U.S. Census, 2000 (ESRI) |
| Distance to nearest hospital * | American Hospital Association Annual Survey Database, 2005 (ESRI) |
| Transportation |  |
| Distance to nearest commercial  airport with >5000 enplanements * | U.S. National Atlas, 2003 (ESRI) |
| Distance to nearest major road * | ESRI, 2002 |
| Natural amenities |  |
| Distance to nearest named river or stream  (square root)* | National Hydrography Dataset, 1:100,000 |
| Distance to nearest lake or pond (square  root)* | National Hydrography Dataset, 1:100,000 |
| Distance to nearest National Park , (Grand  Teton, Rocky Mountain, Wind Cave,  Yellowstone) * | U.S. National Atlas Federal Land Areas, 2004 (ESRI) |
| Distance to nearest National Forest * | within WY: Wyoming Surface and Mineral Ownership, BLM 2010;beyond WY: U.S. National Atlas Federal Land Areas, 2004 |
| Distance to nearest federal or state land | Within WY: Wyoming Surface and Mineral Ownership, BLM 2010; beyond WY: U.S. National Atlas Federal Land Areas, 2004 |
| Proportion of cells with federal or state  land within an 8-km neighborhood * | Within WY: Wyoming Surface and Mineral Ownership, BLM 2010; beyond WY: U.S. National Atlas Federal Land Areas, 2004 |
| Proportion of cells with forested land  within an 8-km neighborhood | Davidson, A., J. Aycrigg, E. Grossmann, J. Kagan, S. Lennartz, S. McDonough, T. Miewald , J. Ohmann, A. Radel, T. Sajwaj, C. Tobalske. 2009. Digital Land Cover Map for the Northwestern United States. Northwest Gap Analysis Project: USGS GAP Analysis Program. http://www.gap.uidaho.edu/Northwest/data.htm. |
| Topography and location |  |
| Elevation | National Elevation Dataset, USGS |
| Topographic position – canyon, ridge,  slope, valley bottom | Used Topographic Slope Position tool within Corridor Designer (Majka et al. 2007) on National Elevation Dataset, USGS. Specifications: 150 cell neighborhood, canyon threshold -10, ridge top threshold 10, slope threshold 6 |
| County * |  |
| Residential and energy development |  |
| Mean 1990 housing density within an 8-  km neighborhood* | U.S. Census, 1990 |
| Mean oil/gas development potential  within an 8-km neighborhood* | Oil and gas potential model presented in this paper |
