# Supplementary material for: Measuring the Effectiveness of Conservation: A Novel Framework to Quantify the Benefits of Sage-Grouse Conservation Policy and Easements in Wyoming
Source: PLoS One. 2013 Jun 24;8(6):e67261. doi: 10.1371/journal.pone.0067261 (PMC3691158; doi:10.1371/journal.pone.0067261)
Supplement: Table S2 — Projected number of new rural housing structures for each county for short-term and long-term scenarios and incorporated towns from which projected housing structures were excluded. (DOCX) [file pone.0067261.s002.docx]

**Table S2**

| **County** | **Number of structures (short-term)** | **Number of structures (long-term)** | **Excluded towns** |
| --- | --- | --- | --- |
| Albany | 490 | 883 | Laramie |
| Big Horn | 795 | 1200 | Greybull, Lovell |
| Campbell | 1462 | 2422 | Gillette, Wright |
| Carbon | 484 | 750 | Hanna, Rawlins, Saratoga |
| Converse | 609 | 907 | Douglas, Glenrock |
| Crook | 874 | 1114 | Hulett, Moorcroft, Sundance |
| Fremont | 1877 | 3158 | Dubois, Lander, Riverton, Shoshoni |
| Goshen | 666 | 972 | Torrington |
| Hot Springs | 372 | 378 | East Thermopolis, Thermopolis |
| Johnson | 766 | 1308 | Buffalo |
| Laramie | 2177 | 4073 | Cheyenne |
| Lincoln | 2097 | 4169 | Afton, Alpine, Diamondville, Kemmerer |
| Natrona | 827 | 2050 | Bar Nunn, Casper, Evansville, Mills |
| Niobrara | 432 | 370 | Lusk |
| Park | 1111 | 1869 | Cody, Meeteetse, Powell |
| Platte | 604 | 874 | Guernsey, Wheatland |
| Sheridan | 963 | 1654 | Sheridan |
| Sublette | 2031 | 4545 | Big Piney, Pinedale |
| Sweetwater | 747 | 1534 | Green River, Rock Springs |
| Teton | 3562 | 4873 | Jackson |
| Uinta | 844 | 1507 | Evanston |
| Washakie | 370 | 538 | Worland |
| Weston | 331 | 554 | Newcastle, Upton |
