# Supplementary material for: Measuring the Effectiveness of Conservation: A Novel Framework to Quantify the Benefits of Sage-Grouse Conservation Policy and Easements in Wyoming
Source: PLoS One. 2013 Jun 24;8(6):e67261. doi: 10.1371/journal.pone.0067261 (PMC3691158; doi:10.1371/journal.pone.0067261)
Supplement: Text S1 — Additional methods text on oil and gas, wind and residential development modeling. (DOCX) [file pone.0067261.s003.docx]

**Text S1**

**Oil and gas development modeling details**

The predictive oil and gas model was developed using Random Forests (RF). RF uses an iterative bootstrap with replacement (64% of data per bootstrap replicate) to construct an ensemble of ‘‘weak learners’’ (CARTs based on a random subsample of data) [[1](#_ENREF_1)]. Prediction is made through a majority vote across the ensemble, not by the familiar rule-set in a traditional CART model and results in both binary and probabilistic continuous model outputs. The derivation of a probabilistic output from a classification-based model was introduced in Evans and Cushman [[2](#_ENREF_2)] as an extension of the original Breiman [[1](#_ENREF_1)] algorithm. It has been shown that the RF algorithm can find signals in noisy data, handle large numbers of predictor variables, avoid over fit, and is invariant to parametric assumptions (e.g. spatial autocorrelation, normality) [[1-3](#_ENREF_1)]. We used a further modified version of RF that addressed the imbalanced data problem [[2](#_ENREF_2)], which is akin to zero inflation in logistic regression. This method iteratively down-samples the majority-class to the approximate size of the minority-class and runs a series of independent models, with fixed parameters, on these random subsets. Because RF is an ensemble learner it is possible to then combine these models into a new ensemble. This avoids validation and prediction bias towards the majority class.

Unfortunately, testing local error for a model generated with Random Forests is not readily feasible. We report on the possible sources of local error as an alternative. One likely missing covariate is the underlying geologic formation data, which was unavailable contiguously across the study area. However, we remain confident in this model’s predictions for the scale of the application presented. A study validating climate models concluded that model error decreases as spatial extent and scale increases [[4](#_ENREF_4)]; similar multivariate spatial models will likely have the greatest error at the smallest spatial scales, and that error decreases with regional or larger scale predictions. Consequently, we have higher confidence in the global calculations of range-wide species impacts than estimates at any given location. Great caution should be used for local scale interpretations.

We mapped legal constraints to oil and gas development using data acquired from the GIS section of the Wyoming BLM website including “no surface occupancy”, withdrawals, and Wilderness Study Areas. Seasonal and timing stipulations were not included because not all these data are available consistently across our study area. We also included other private, federal and state protected lands such as Nature Conservancy Preserves, US Fish and Wildlife Service Refuges, Wyoming Game & Fish Habitat Management Areas, which correspond to International Union for the Conservation of Nature (IUCN) protected category of five or lower.

**Wind energy development modeling details**

Maximum entropy was used to model potential wind development. Maximum entropy is nonparametric and insensitive to multicollinearity and has consistently been shown to be an accurate and robust algorithm, particularly with small sample sizes. Maxent allows the user to define the method used to fit the predictor layers, and to avoid overfitting [[5](#_ENREF_5)] we selected only linear and quadratic features. For the primary model, the order of variable importance was wind power class (55.6% contribution), topographic position (24.9% contribution), and slope (19.4% contribution). We assigned a value of zero to model output values occurring below the logistic threshold of 0.314 (maximum training sensitivity plus specificity), and then scaled the new wind resource potential values from 0-1.

We adjusted the Maxent-generated wind resource potential raster dataset to reflect expected short-term development and legal or operational constraints. Short-term development was represented by the kernel density (15-km search radius) of existing meteorological towers [[6](#_ENREF_6)], distance (log) to proposed electrical transmission lines with capacity of at least 230 kV [[7](#_ENREF_7)], proposed wind farm boundaries [[8](#_ENREF_8)] and land tenure [[9](#_ENREF_9)]. Meteorological towers represent locations where developers are currently evaluating wind resources on the ground, and new developments will likely be located near new transmission lines since existing lines are currently at capacity. Many proposed wind farms are likely to be developed, so these locations were weighted most heavily to reflect where wind development is expected in the near term. Proposed wind farms in Sweetwater and Uinta counties were assigned a weight of 2, compared with 1 in other counties, due to lower wind resource potential in those areas but high likelihood that these specific farms will be developed. Privately-owned lands were weighted most heavily (value =1), followed by state (value =0.8) and federal lands (value =0.5, except for Fish and Wildlife Service lands, where value = 0), because there are fewer regulations governing development on private lands and to date nearly all Wyoming wind turbines have been constructed on private lands. The datasets related to short-term development were each on a scale from 0-1, with exception of proposed wind farms which were assigned values of one or two. All of the scaled raster datasets, except land tenure, were added to wind resource potential raster, and then the result of the addition was multiplied by the scaled land tenure raster. The raster dataset was rescaled from 0-1 following these calculations. Next we assigned raster cells having legal or operational constraints with zero probability for wind development. Legally protected lands included those in International Union for Conservation of Nature (IUCN) categories one through five, which include wilderness areas, wildlife refuges, and conservation easements (details in [[10](#_ENREF_10)], as well as lands managed by the Bureau of Land Management that have “No Surface Occupancy” stipulations. Wind turbines cannot be constructed within airport runway air space, which was represented by digitizing runways of all municipal airports and applying a 1524-m buffer per federal guidelines [[11](#_ENREF_11)]. Urban areas [[12](#_ENREF_12)], mountainous areas above 2743-m elevation, and lakes (National Hydrography Dataset) were also excluded from potential development. Finally, the dataset was smoothed to remove isolated high-potential raster cells not likely to be developed and scaled by applying a square root function. The wind development potential raster dataset was developed at 100-m resolution and then averaged to a 1-km resolution.

**Residential development modeling details**

The predictive model’s response variable, change in housing density, was calculated from 1990 and 2010 block-level housing unit data obtained from the US census. For each date, we clipped each census block to the extent of private lands using statewide ownership data [[9](#_ENREF_9)], but used county parcel data for Teton and Carbon counties, occurring within that polygon. We then calculated the number of housing units per hectare for each modified block and maximum density was constrained to 100 where values were inflated due to GIS boundary errors. For both years, a 10-m resolution raster dataset was created to capture the patterns in small census blocks. Mean housing density was calculated from the 10-m dataset using a 1-km window and the output resampled to 1-km resolution. Change was calculated by subtracting the 1990 raster from the 2010 raster and negative values were recoded as zero. The 16 predictor variables are described in Table S1.

We fit several Random Forest models and applied the model with the lowest error for our forecasting. The Random Forests approach is described in detail above in the section on oil and gas development modeling details. We first fit a regression model, where the response variable was the change in housing density, but this model explained less than 4% of the variation and was not considered further. Second, we fit six variations of classification models including binary (no change or change/increase), and three to five classes of change in housing density as the response variable. For each case of the model, we applied the Model Improvement Ratio [[13](#_ENREF_13)] to compare subsets of variables and identify the most parsimonious model having the lowest error. We ran 300 bootstrap replicates and for each replicate withheld 36% of the data as an OOB sample. A binary model was selected as the best-fitting model, based on OOB error (11.7%) and class-level error .Variables used in the selected model are shown in Table S1. We used the selected binary model to predict a probabilistic output [[2](#_ENREF_2)].

The numbers of projected housing structures, by county, for the short and long-term scenarios are presented in Table S2.

Existing housing structures have not been mapped in Wyoming, so we created this dataset using 2010 census data. A number of housing units is associated with each census block, and these data were constrained to the spatial extent of private lands, as described previously. To account for multi-unit dwellings, we determined the proportion of single- and multi-unit dwellings for each county using data from the American Community Survey. We applied these proportions to adjust housing unit numbers downward in census blocks occurring within the boundaries of incorporated towns. We did not make this adjustment outside of towns, because few multi-unit housing structures occur in rural areas. Density was constrained to 15 structures per hectare in urban areas where GIS boundary errors resulted in artificially inflated values. Random points were generated within the boundaries of each census block, at least 11 m apart, to reflect the number of structures occurring there. The spatial accuracy of housing locations decreases as census blocks increase in size. Over 78% of the census blocks are 100 ha or smaller, and therefore most points have adequate precision for our modeling at 1-km resolution. Fewer than 2% of the census blocks containing houses are greater than 1000 ha in size.

References

1. Breiman L (2001) Random forests. Machine Learning 45: 5-32.

2. Evans JS, Cushman SA (2009) Gradient modeling of conifer species using random forests. Landscape Ecol 24: 673-683.

3. Cutler RD, Edwards TC, Beard KH, Cutler A, Hess KT, et al. (2007) Random forests for classification in ecology. Ecology 88: 2783-2792.

4. Masson D, Reto K (2011) Spatial-Scale Dependence of Climate Model Performance in the CMIP3 Ensemble. Journal of Climate 24: 2680-2692.

5. Phillips SJ, Dudik M (2008) Modeling of species distributions with Maxent: new extensions and a comprehensive evaluation. Ecography 31: 161-175.

6. Wyoming Game and Fish Department (2010) Met tower spatial locations. Cheyenne, Wyoming: Wyoming Game and Fish Department.

7. Ventyx Energy (2010) Electric transmission lines. December 2010 ed: Ventyx.

8. Wyoming Department of Environmental Quality (2011) GIS boundaries of wind farms regulated by Industrial Siting Council. June 2011 ed. Cheyenne, Wyoming: Wyoming Industrial Siting Council.

9. Bureau of Land Management (2010) Wyoming surface and mineral ownership. Available: <http://www.blm.gov/wy/st/en/resources/public_room/gis/datagis.html>. Accessed 12 December 2010

10. Pocewicz A, Copeland HE, Buchmann M (2009) The state of habitat protection in Wyoming. Lander, Wyoming: The Nature Conservancy.

11. Federal Aviation Administration (2010) Safe, efficient use and preservation of the navigable airspace. Washington, DC: Federal Register (75 FR 42296).

12. Wyoming Department of Revenue (2011) Wyoming incorporated town boundaries. Available: <http://revenue.state.wy.us>. Accessed January 2011

13. Murphy MA, Evans JS, Storfer A (2010) Quantifying *Bufo boreas* connectivity in Yellowstone National Park with landscape genetics. Ecology 91: 252-261.
